# Supplementary material for: BatmanNet: bi-branch masked graph transformer autoencoder for molecular representation
Source: Brief Bioinform. 2023 Nov 29;25(1):bbad400. doi: 10.1093/bib/bbad400 (PMC10783874; doi:10.1093/bib/bbad400)
Supplement: supplementary_information_bbad400 [file supplementary_information_bbad400.pdf]

Supplementary Section 1: Details about Molecular Datastes

1.1 Downstream task datasets.

In this paper, we have evaluated our method on a wide range of downstream drug discovery tasks, including molecular properties prediction, drug-drug interaction (DDI) prediction, and drug-target interaction (DTI) prediction.

Molecular properties prediction datasets

- **BBBP** [69] provides records of whether a compound carries the permeability property of penetrating the blood-brain barrier.
- **SIDER** [70] is a database of marketed drugs and adverse drug reactions (ADR), grouped into 27 system organ classes.
- **ClinTox** [71] compares drugs approved through the FDA and drugs eliminated due to toxicity during clinical trials.
- **BACE** [72] provides quantitative blinding results for a set of inhibitors of human β-secretase 1 (BACE-1).
- **Tox21** [73] is a public database measuring the toxicity of compounds on 12 different targets, including nuclear receptors and stress response.
- **ToxCast** [74] providing toxicology data for 8615 compounds based on in vitro high-throughput screening.
- **FreeSolv** [75] provides experimental and calculated hydration free energy of small molecules in water. The calculated values are derived from alchemical free energy calculations using molecular dynamics simulations.
- **ESOL** [76] is a small dataset consisting of water solubility data for 1128 compounds.
- **Lipo** [77] is curated from the ChEMBL database, which is an important feature of drug molecules that affects both membrane permeability and solubility and provides experimental results of octanol/water distribution coefficient (log D at pH 7.4) of 4200 compounds.
- **QM7** [78] is a subset of the GDB-13 database, a database of nearly 1 billion stable and synthetically accessible organic molecules, containing up to seven “heavy” atoms (C, N, O, S).
- **QM8** [79] are applied to a collection of molecules that include up to eight heavy atoms (also a subset of the GDB-17 database). It contains computer-generated quantum mechanical properties.

DDI prediction datasets

- **BIOSNAP** [67] that consists of 1322 approved drugs with 41520 labeled DDIs, obtained through drug labels and scientific publications.
- **TWOSIDES** [68] contains side effects caused by the combination of drugs, which contains 548 drugs and 48584 pair-wise drug-drug interactions.

DTI prediction datasets

- **Human** and **C.lelgan**, created by Liu et al., include highly credible negative samples of compound-protein pairs by using a systematic framework. Positive samples of the datasets were retrieved from DrugBank 4.1 and Matador. We used a balanced dataset with a ratio of 1:1 of positive and negative samples following Tsubaki et al. [65] and MPG []li2021effective.

Dataset Splitting. In most machine learning applications, the traditional method of random splitting is used to split the dataset. However, in practice, the molecules used for testing may be different from the training molecules in scaffold structure, i.e., out-of-distribution prediction and the way of random splitting is ideal for simulating real-world situations. Unlike random splitting, scaffold splitting splits the data set into different subsets according to the molecule's structure. This challenging but reliable splitting method tests the model's generalization ability outside the distribution (out-of-distribution generalization). We use scaffold splitting to split the dataset into training, validation, and test sets at a ratio of 8:1:1 in each downstream task.

Supplementary Section 2: Implementation Details

2.1 Atom and bond features

We use RDKit to extract the atom and bond features as the input of GNN and the reconstruction target of BatmanNet. Table S1 shows the atom and bond features we used in BatmanNet.

2.2 Pre-training Details

We use the Adam optimizer with an initial learning rate of 0.0002 and L2 weight decay for 10^{-7}. We train the model for 20 epochs. The learning rate warmed over the first epoch and decreased exponentially from 0.0004 to 0.0001. Table S2 demonstrates all the hyper-parameters of the pre-training model, Among these, the parameter mask_ratio is chosen based on the experimental results in Table S6, and the batch_size is selected according to the GPU memory. The parameters depth, num_enc_mt_block, num_dec_mt_block, num_dec_mt_block, and num_attn_head are determined based on the model parameter amount, convergence situation and experience.

2.3 Fine-tuning Details

Fine-tuning implementation

We only use BatmanNet's encoder for downstream tasks. Unlike the pre-training, where the model input is an incomplete molecule, the inputs of downstream tasks are complete molecules without masking. After N GNN-Attention blocks, both branches of BatmanNet's encoder perform Node Aggregation, producing two node representations $\mathbf{m}_v^{\text{node-branch}}$ and $\mathbf{m}_v^{\text{edge-branch}}$ as follows:

$$\mathbf{m}_v^{\text{node-branch}} = \sum_{u \in \mathcal{N}_v} \bar{\mathbf{h}}_u, \tag{15}$$

$$\mathbf{m}_v^{\text{edge-branch}} = \sum_{u \in \mathcal{N}_v \setminus w} \bar{\mathbf{h}}_{uv}, \tag{16}$$

where $\bar{\mathbf{h}}_u$ and $\bar{\mathbf{h}}_{uv}$ are the hidden states of the GNN-Attention blocks of node-branch and edge-branch. Then we also apply a single long-range residual connection to concatenate $\mathbf{m}_v^{\text{node-branch}}$ and $\mathbf{m}_v^{\text{edge-branch}}$ with initial node features and edge features, respectively. Finally, we transform the two embeddings $\mathbf{m}_v^{\text{node-branch}}$ and $\mathbf{m}_v^{\text{edge-branch}}$ through Feed Forward layers and LayerNorm to generate the final two embeddings output for downstream tasks.

Through the above process, given a molecule G_i and the corresponding label $\boldsymbol{y}_i$, BatmanNet's encoder can generate two node embeddings, $\mathbf{H}_i^{\text{node-branch}}$ and $\mathbf{H}_i^{\text{edge-branch}}$, from the node branch and the edge branch, respectively. Following GROVER [18], we feed these two node embeddings into

a shared self-attentive READOUT function to generate two graph-level embeddings, $\boldsymbol{g}^{\text{node-branch}}$ and $\boldsymbol{g}^{\text{edge-branch}}$. They are both obtained by:

$$\mathbf{S} = \text{softmax}\left(\mathbf{W}_2 \tanh\left(\mathbf{W}_1 \mathbf{H}^\top\right)\right), \tag{17}$$

$$\boldsymbol{g} = \text{Flatten}(\mathbf{SH}), \tag{18}$$

where $\mathbf{W}_1 \in \mathbb{R}^{d_{\text{attn\_hidden}} \times d_{\text{hidden\_size}}}$ and $\mathbf{W}_2 \in \mathbb{R}^{d_{\text{att\_out}} \times d_{\text{att\_hidden}}}$ are two weight matrix.

Molecular properties prediction. After getting two graph-level embeddings $\boldsymbol{g}^{\text{node-branch}}$ and $\boldsymbol{g}^{\text{edge-branch}}$, we apply a Feed Forward layer for both branches to get predictions $\boldsymbol{p}_i^{\text{node-branch}}$ and $\boldsymbol{p}_i^{\text{edge-branch}}$.

$$\boldsymbol{p}_i^{\text{node-branch}} = f\left(\boldsymbol{W}\boldsymbol{g}^{\text{node-branch}} + b\right), \tag{19}$$

$$\boldsymbol{p}_i^{\text{edge-branch}} = f\left(\boldsymbol{W}\boldsymbol{g}^{\text{edge-branch}} + b\right), \tag{20}$$

DDI prediction. In the DDI prediction task, the input is a pair of molecules, which are encoded into two sets of graph-level embeddings ($\boldsymbol{g}_1^{\text{node-branch}}$ and $\boldsymbol{g}_1^{\text{edge-branch}}$, $\boldsymbol{g}_2^{\text{node-branch}}$ and $\boldsymbol{g}_2^{\text{edge-branch}}$) by the encoder of BatmanNet, respectively. The predictions $\boldsymbol{p}_i^{\text{node-branch}}$ and $\boldsymbol{p}_i^{\text{edge-branch}}$ are calculated by:

$$\boldsymbol{p}_i^{\text{node-branch}} = f\left(\boldsymbol{W}\boldsymbol{g}_{pair}^{\text{node-branch}} + b\right), \tag{21}$$

$$\boldsymbol{p}_i^{\text{edge-branch}} = f\left(\boldsymbol{W}\boldsymbol{g}_{pair}^{\text{edge-branch}} + b\right), \tag{22}$$

$$\boldsymbol{g}_{pair}^{\text{node-branch}} = \text{Concat}\left(\boldsymbol{g}_1^{\text{node-branch}}, \boldsymbol{g}_2^{\text{node-branch}}\right), \tag{23}$$

$$\boldsymbol{g}_{pair}^{\text{edge-branch}} = \text{Concat}\left(\boldsymbol{g}_1^{\text{edge-branch}}, \boldsymbol{g}_2^{\text{edge-branch}}\right), \tag{24}$$

DTI prediction. In this study, following MPG [13], we adapt Tsubaki et al.'s DTI framework to accomplish the DTI prediction task by replacing the molecular encoder (GNN) with our BatmanNet's encoder. The protein sequence encoder is a CNN model. It uses the attention mechanism to derive the protein sequence representation $\boldsymbol{y}_p$. Given a set of hidden vectors of sub-sequences in a protein $S = (s_1, s_2, ..., s_n)$, the $\boldsymbol{y}_p$ is calculated by:

$$\boldsymbol{y}_p = \sum_i^n (\alpha_i h_i), \tag{25}$$

$$\boldsymbol{\alpha}_i = \sigma\left(\boldsymbol{h}_m^T \boldsymbol{h}_i\right), \tag{26}$$

$$\boldsymbol{h}_m = f\left(\boldsymbol{W}\boldsymbol{g}_m + b\right), \tag{27}$$

$$\boldsymbol{h}_i = f\left(\boldsymbol{W}s_i + b\right) \tag{28}$$

where $\boldsymbol{g}_m$ is a molecular vector and the weight for s_i considering $\boldsymbol{g}_m$. $\boldsymbol{W}$ is the pearned weight matrix, b is the bias vector, and $\boldsymbol{\alpha}_i$ is the attention weights.

Like the molecular properties prediction, we get two molecular embeddings $\boldsymbol{g}^{\text{node-branch}}$ and $\boldsymbol{g}^{\text{edge-branch}}$ by the encoder of BatmanNet. Then we get two protein embeddings $\boldsymbol{y}_p^{\text{node-branch}}$ and $\boldsymbol{y}_p^{\text{edge-branch}}$ by formula (25). The predictions $\boldsymbol{p}_i^{\text{node-branch}}$ and $\boldsymbol{p}_i^{\text{edge-branch}}$ are calculated by:

$$\boldsymbol{p}_i^{\text{node-branch}} = f\left(\boldsymbol{W}\boldsymbol{y}_{pair}^{\text{node-branch}} + b\right), \tag{29}$$

$$\boldsymbol{p}_i^{\text{edge-branch}} = f\left(\boldsymbol{W}\boldsymbol{y}_{pair}^{\text{edge-branch}} + b\right), \tag{30}$$

$$\boldsymbol{y}_{pair}^{\text{node-branch}} = \text{Concat}\left(\boldsymbol{g}^{\text{node-branch}}, \boldsymbol{y}_p^{\text{node-branch}}\right), \tag{31}$$

$$\boldsymbol{y}_{pair}^{\text{edge-branch}} = \text{Concat}\left(\boldsymbol{g}^{\text{edge-branch}}, \boldsymbol{y}_p^{\text{edge-branch}}\right), \tag{32}$$

The final loss of downstream tasks consists of the supervised loss $\mathcal{L}_{\text{sup}}$ and the disagreement loss [80] $\mathcal{L}_{\text{diss}}$, where the disagreement loss is to train the two predictions to be consistent.

$$\mathcal{L}_{\text{fine-tune}} = \mathcal{L}_{\text{sup}} + \mathcal{L}_{\text{diss}}, \tag{33}$$

$$\mathcal{L}_{\text{sup}} = \mathcal{L}\left(\boldsymbol{p}_i^{\text{node-branch}}, \boldsymbol{y}_i\right) + \mathcal{L}\left(\boldsymbol{p}_i^{\text{edge-branch}}, \boldsymbol{y}_i\right), \tag{34}$$

$$\mathcal{L}_{\text{diss}} = \left\|\boldsymbol{p}_i^{\text{node-branch}} - \boldsymbol{p}_i^{\text{edge-branch}}\right\|_2. \tag{35}$$

The Fine-tuning Hyperparams

For each task, we try different hyper-parameter combinations via random search to find the best results Table S3 shows all the hyper-parameters of the fine-tuning model.

Supplementary Section 3: Supplementary Experimental Results

3.1 Additional experiments

We additionally conduct experiments on molecular properties prediction following the same experimental settings used in GEM [20]. As shown in Table S5, BatmanNet achieves state-of-the-art performance on 7 out of 11 datasets, with an overall relative improvement of 1.1% compared to the previous SOTA results on all the datasets (1.8% on classification tasks and 0.4% on regression tasks). Note that, the results of GraphMAE are from [47], and the results of other baselines are directly copied from [20].

3.2 Details of The Effect of Different Masking Ratio

Table S6 shows the specific experimental results of the BatmanNet pre-trained with different masking ratios (ranging from 0.1 to 0.9) on eight benchmark datasets. Figure S1 shows the influence of the masking ratio on each benchmark dataset, respectively. The results show that setting the masking ratio to 60% achieves the best prediction performance, demonstrating the consistency of our BatmanNet's performance on various datasets.

Table S1. Atom and Bond features.

	Features	Size	Description
Atom	Atom type	23	The atom type (e.g., C, N, O), by atomic number
	Number of H	6	The number of bonded hydrogen atoms
	Charge	5	The formal charge of the atom
	Chirality	4	The chiral-tag of the atom
	Is-aromatic	1	Whether the atom is part of an aromatic system or not
Bond	Bond type	5	The bond type (e.g., single, double, triple et al.)
	Stereo	6	The stereo-configuration of the bond

Table S2. The pre-training hyper-parameters.

Hyper-parameter	Value	Description
batch_size	32	The input batch_size
hidden_size	100	The hidden_size of encoder and decoder
depth	3	The number of GNN layers in GNN-Attention block
num_enc_mt_block	6	The number of the GNN-Attention block in encoder
num_dec_mt_block	2	The number of the GNN-Attention block in decoder
num_attn_head	2	The number of attention heads in the GNN-Attention block
mask_ratio	0.6	The mask ratio
init_lr	0.0002	The initial learning rate of Noam learning rate schedular
max_lr	0.0004	The maximum learning rate of Noam learning rate schedular
final_lr	0.0001	The final learning rate of Noam learning rate schedular

Table S3. The fine-tuning hyper-parameters.

Hyper-parameter	Value	Description
batch_size	32	The input batch_size
ffn_hidden_size	200	The hidden_size of MLP layers
ffn_num_layer	2	The number of MLP layers
attn_hidden	200	The hidden_size for the self-attentive readout
attn_out	2	The number of output heads for the self-attentive readout
dist_coff	0.1	The coefficient of the disagreement loss
init_lr	max_lr / 10	The initial learning rate of Noam learning rate schedular
max_lr	0.0001 0.001	The maximum learning rate of Noam learning rate schedular
final_lr	max_lr / (5-10)	The final learning rate of Noam learning rate schedular

Table S4. The table illustrates the pre-training dataset size and model size for BatmanNet and a series of advanced baselines, along with their average AUC across all classification datasets for molecular property prediction.

Model	Pre-training Data Size (M)	Model Size (M)	AVG-AUC /%
GraphMAE	2	-	78.90
GROVERbase	11	40	82.28
GROVERlarge	11	100	83.40
KPGT	2	-	82.53
MPG	11	55	84.18
GEM	20	-	85.15
BatmanNet	0.25	2.6	84.78

Table S5. Overall performance for classification tasks and regression tasks of molecular properties prediction following the same experimental settings used in GEM

Methods	Classification (AUC-ROC)								
Dataset	BACE	BBBP	Clin Tox	SIDER	Tox21	ToxCast	HIV	MUV	Avg
#molecules	1513	2039	1478	1427	7831	8575	41127	93087	-
#tasks	1	1	2	27	12	617	1	17	-
D-MPNN	$0.809_{(0.006)}$	$0.710_{(0.003)}$	$0.906_{(0.006)}$	$0.570_{(0.007)}$	$0.759_{(0.007)}$	$0.655_{(0.003)}$	$0.771_{(0.005)}$	$0.786_{(0.014)}$	0.746
AttentiveFP	$0.784_{(0.022)}$	$0.643_{(0.018)}$	$0.847_{(0.003)}$	$0.606_{(0.032)}$	$0.761_{(0.005)}$	$0.637_{(0.002)}$	$0.757_{(0.014)}$	$0.766_{(0.015)}$	0.735
N-GramRF	$0.779_{(0.015)}$	$0.697_{(0.006)}$	$0.775_{(0.040)}$	$0.668_{(0.007)}$	$0.743_{(0.004)}$	-	$0.772_{(0.001)}$	$0.769_{(0.007)}$	-
N-GramXGB	$0.791_{(0.013)}$	$0.691_{(0.008)}$	$0.875_{(0.027)}$	$0.655_{(0.007)}$	$0.758_{(0.009)}$	-	$0.787_{(0.004)}$	$0.748_{(0.002)}$	-
PretrainGNN	$0.845_{(0.007)}$	$0.687_{(0.013)}$	$0.726_{(0.015)}$	$0.627_{(0.008)}$	$0.781_{(0.006)}$	$0.657_{(0.006)}$	$0.799_{(0.007)}$	$0.813_{(0.021)}$	0.742
GROVERbase	$0.826_{(0.007)}$	$0.700_{(0.001)}$	$0.812_{(0.030)}$	$0.648_{(0.006)}$	$0.743_{(0.001)}$	$0.654_{(0.004)}$	$0.625_{(0.009)}$	$0.673_{(0.018)}$	0.710
GROVERlarge	$0.810_{(0.014)}$	$0.695_{(0.001)}$	$0.762_{(0.037)}$	$0.654_{(0.001)}$	$0.735_{(0.001)}$	$0.653_{(0.005)}$	$0.682_{(0.011)}$	$0.673_{(0.018)}$	0.708
GraphMAE	$0.831_{(0.009)}$	$0.720_{(0.006)}$	$0.823_{(0.012)}$	$0.603_{(0.011)}$	$0.755_{(0.006)}$	$0.641_{(0.003)}$	$0.772_{(0.010)}$	$0.763_{(0.024)}$	0.739
GEM	$0.856_{(0.011)}$	$0.724_{(0.004)}$	$0.901_{(0.013)}$	$0.672_{(0.004)}$	$0.781_{(0.001)}$	$0.692_{(0.004)}$	$0.806_{(0.009)}$	$0.817_{(0.005)}$	0.781
BatmanNet	$\mathbf{0.861_{(0.028)}}$	$\mathbf{0.838_{(0.005)}}$	$0.897_{(0.012)}$	$0.659_{(0.003)}$	$\mathbf{0.792_{(0.003)}}$	$\mathbf{0.718_{(0.007)}}$	$\mathbf{0.812_{(0.009)}}$	$0.784_{(0.014)}$	**0.795**

Methods	Regression (RMSE)			
Model	ESOL	FreeSolv	Lipo	Avg
#molecules	1128	642	4200	-
#tasks	1	1	1	-
D-MPNN	$1.050_{(0.008)}$	$2.082_{(0.082)}$	$0.683_{(0.016)}$	1.272
AttentiveFP	$0.877_{(0.029)}$	$2.073_{(0.183)}$	$0.721_{(0.001)}$	1.224
N-Gram_RF	$1.074_{(0.107)}$	$2.688_{(0.085)}$	$0.812_{(0.028)}$	1.525
N-Gram_XGB	$1.083_{(0.082)}$	$5.061_{(0.744)}$	$2.072_{(0.030)}$	2.739
PretrainGNN	$1.100_{(0.006)}$	$2.764_{(0.002)}$	$0.739_{(0.003)}$	1.534
GROVERbase	$0.983_{(0.090)}$	$2.176_{(0.052)}$	$0.817_{(0.008)}$	1.325
GROVERlarge	$0.895_{(0.017)}$	$2.272_{(0.051)}$	$0.823_{(0.010)}$	1.330
GEM	$0.798_{(0.029)}$	$1.877_{(0.094)}$	$0.660_{(0.008)}$	1.112
BatmanNet	$\mathbf{0.792_{(0.013)}}$	$\mathbf{1.802_{(0.006)}}$	$0.729_{(0.015)}$	**1.108**

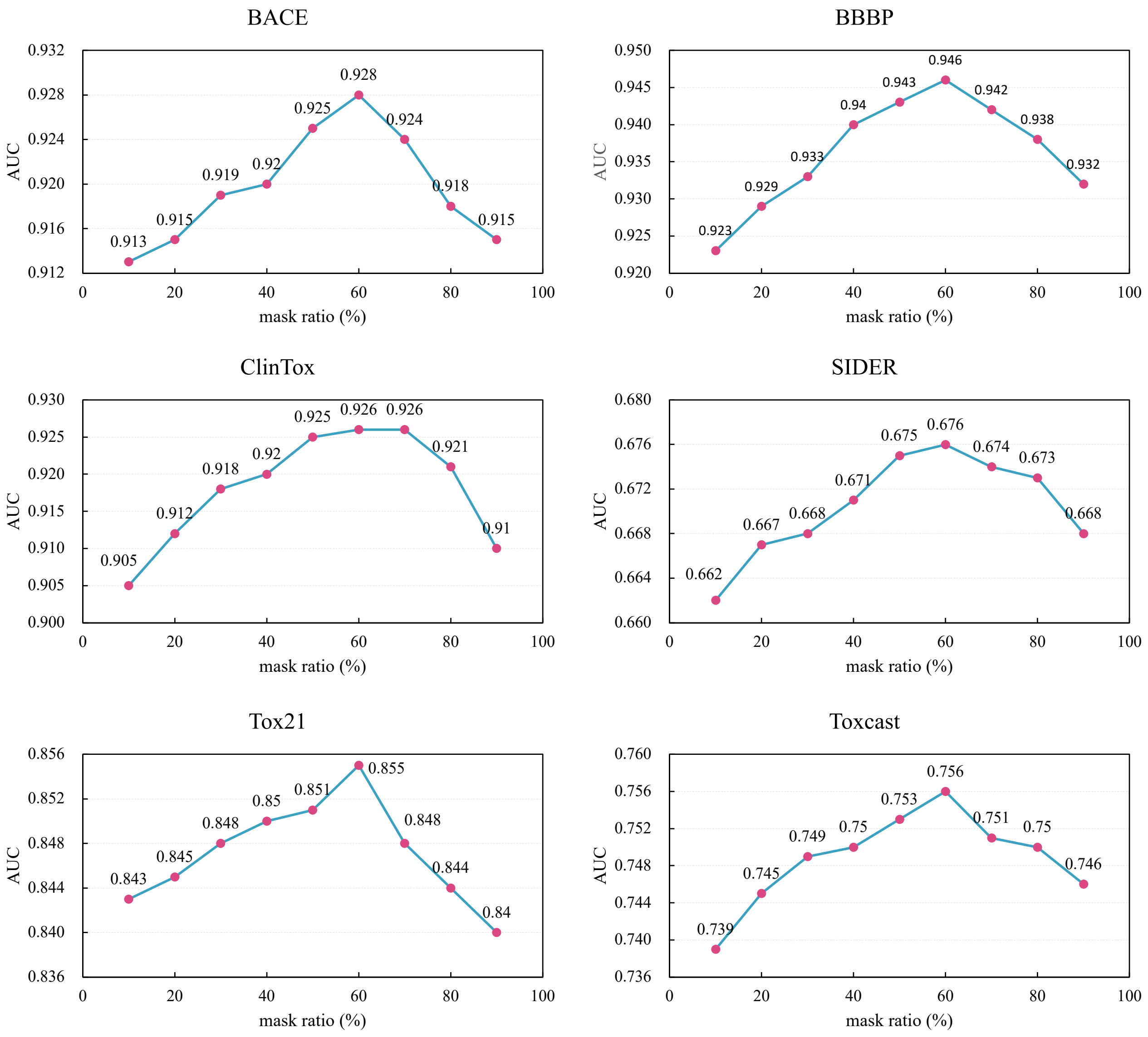

Fig. S1. The influence of the masking ratio on each benchmark dataset.

Table S6. The experimental results of the BatmanNet pre-trained with different masking ratios (ranging from 0.1 to 0.9) on eight benchmark datasets. We report the mean (and standard deviation) AUC for each dataset of three random seeds with scaffold splitting.

Ratio	BBBP	SIDER	ClinTox	BACE	Tox21	ToxCast	Avg
0.1	$0.923_{(0.032)}$	$0.662_{(0.015)}$	$0.905_{(0.028)}$	$0.913_{(0.007)}$	$0.843_{(0.014)}$	$0.739_{(0.011)}$	0.831
0.2	$0.929_{(0.027)}$	$0.667_{(0.003)}$	$0.912_{(0.012)}$	$0.915_{(0.006)}$	$0.845_{(0.009)}$	$0.745_{(0.009)}$	0.836
0.3	$0.933_{(0.018)}$	$0.668_{(0.003)}$	$0.918_{(0.025)}$	$0.919_{(0.013)}$	$0.848_{(0.017)}$	$0.749_{(0.007)}$	0.839
0.4	$0.940_{(0.011)}$	$0.671_{(0.006)}$	$0.920_{(0.028)}$	$0.920_{(0.014)}$	$0.850_{(0.014)}$	$0.750_{(0.009)}$	0.842
0.5	$0.943_{(0.019)}$	$0.675_{(0.004)}$	$0.925_{(0.025)}$	$0.925_{(0.014)}$	$0.851_{(0.013)}$	$0.753_{(0.008)}$	0.845
0.6	$\mathbf{0.946_{(0.007)}}$	$\mathbf{0.676_{(0.004)}}$	$\mathbf{0.926_{(0.015)}}$	$\mathbf{0.928_{(0.015)}}$	$\mathbf{0.855_{(0.013)}}$	$\mathbf{0.756_{(0.009)}}$	**0.848**
0.7	$0.942_{(0.008)}$	$0.674_{(0.004)}$	$\mathbf{0.926_{(0.011)}}$	$0.924_{(0.016)}$	$0.848_{(0.012)}$	$0.751_{(0.007)}$	0.844
0.8	$0.938_{(0.012)}$	$0.673_{(0.004)}$	$0.921_{(0.288)}$	$0.918_{(0.016)}$	$0.844_{(0.014)}$	$0.750_{(0.008)}$	0.841
0.9	$0.932_{(0.020)}$	$0.668_{(0.005)}$	$0.910_{(0.022)}$	$0.915_{(0.015)}$	$0.840_{(0.015)}$	$0.746_{(0.011)}$	0.835
